# The influence of strong social ties on the choice of long-term care model for middle-aged and older adults in China

**DOI:** 10.3389/fpubh.2023.1112422

**Published:** 2023-05-12

**Authors:** Tao Jing, Xinmeng Zhao, Huixia Xing, Chuang Bao, Lanxi Zhan

**Affiliations:** School of Insurance, University of International Business and Economics, Beijing, China

**Keywords:** China, strong social relationship, population aging, long-term care, SIRS Contagion Model

## Abstract

With the acceleration of population aging in China, the care of middle-aged and older adults has become the focus of social attention. As China is an “acquaintance society,” strong social relations play an important role in residents’ access to information and resource allocation, which has an impact on the choice of long-term care models for middle-aged and older adults. Therefore, based on the 19,728 samples from the 2018 CHARLS Phase I data, an empirical analysis was conducted using a logistic dichotomous model, which showed that both kinship (*p* < 0.01) and friendship (p < 0.01) among social relationships positively influenced the choice of social care models for middle-aged and older adults. The analysis of the heterogeneity showed that the choice of long-term care models was significantly influenced by strong social relationships in the central and western regions and rural areas (*p* < 0.01). On the basis of this, the transmission mechanism of strong social relationships was further analyzed in conjunction with the further construction of the SIRS Contagion Model, which was used to describe the information transmission process. The results showed that residents who attached importance to strong social ties were more likely to incur medical transfer expenditure, thus further increasing their own demand for the formal care model. The policy implications of this study are to promote the coordinated development of long-term formal care and strong social relations, to further promote the socialization of the formal care model while relying on traditional family care, and to build a multi-level and diversified long-term care system for middle-aged and older adults.

## 1. Introduction

**Figure 1:**
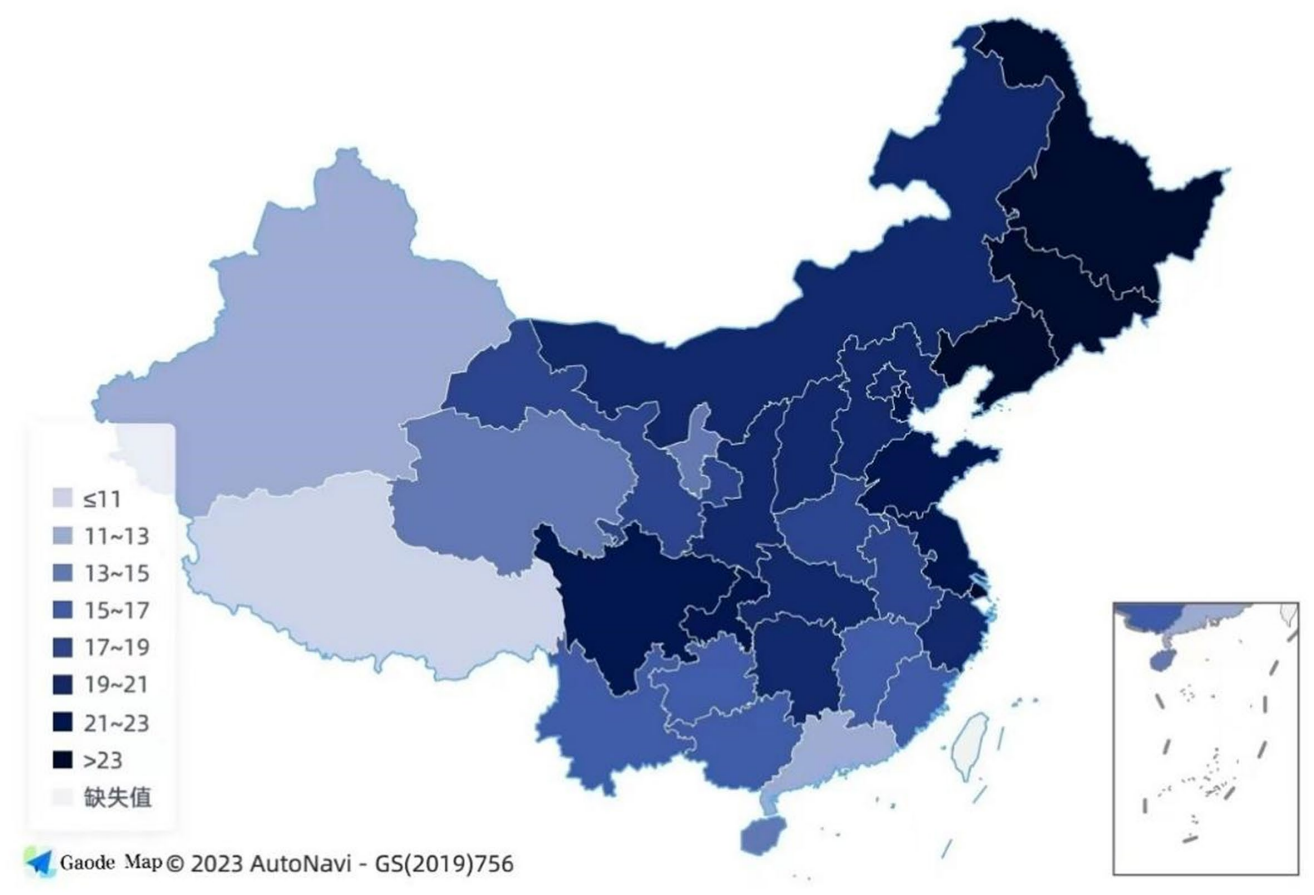
The proportion of older adults aged 60 and above in each province of China.

**Figure 2:**
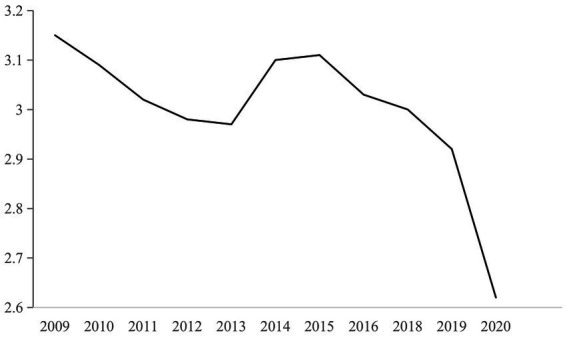
Trends in household size in China.

At present, China’s aging population is growing rapidly and the number of senior citizens is increasing sharply (see Figure 1). According to the results of the seventh national census, by the end of 2020, China had 260 million people aged 60 and above, accounting for 18.70% of the total population. Among them, the proportion of people aged 65 or above reached 13.50%, an increase of 2.51 and 2.72 percentage points^1^ compared with 2010. At the same time, the rate of disability among older adults has also risen sharply. The survey showed that the number of older adults with disabilities has exceeded 42 million, with about one in every six older adults unable to take care of themselves.^2^ In recent years, the family structure tends to be smaller, with fewer children^3^ (see Figure 2), and the dependency ratio of the older adult population has risen to 19.70%. In one interview about older adults, a child of older adults said: “The older adult in the family have changed from cheerful to introverted after becoming disabled, believing that caring for themselves has put pressure on their children, and have even thought of committing suicide.” Due to the rising rates of disability and senior support, such problems have gradually attracted extensive social attention. How to properly deal with the failure of care of middle-aged and older adults in China has become one of the “hot” issues.

In the face of the heavy care problems of the aging society, social home care, institutional care, and other formal care methods continue to rise, but overall older adult care still presents a “9,073” pattern.^4^ In recent years, China has issued a series of long-term care systems and policies for middle-aged and older adults: in 2016, China has implemented long-term care trial projects in Qingdao, Nantong, and other locations to better address the demands of day care for older adults with disabilities. In 2019, the Chinese government issued the Medium and Long Term Plan for National Active Response to Aging Population, which set the goal of “building high-quality supply of older adults care services.”^5^ In 2020, the state again promulgated the Guiding Opinions on Expanding the Trial of the Long-term Care Insurance System to further expand the scope of the trial on the basis of the original pilot cities. In November 2021, the State Council of the People’s Republic of China issued the Opinions on Strengthening Work on Aging in the New Era, proposing to improve the old-age service system and strengthen long-term care services and security for older adults with disabilities.^6^ Although the long-term care system has made certain achievements in China, the development of socialized and diversified long-term care services still has a realistic dilemma. From the perspective of traditional culture, Confucianism has been rooted in our social structure for a long time and the traditional family pension model with “raising children for the old” as the main concept is the mainstream position (1). However, with the continuous improvement of economic levels, household sizes are shrinking and the smaller family structure of “421”^7^ makes the traditional family pension model difficult to sustain. A socialized formal care model that can address the issues of population aging and aging disability should be introduced in this situation (2).

In the face of the increasingly diversified care model, how middle-aged and older adults choose what kind of care model is affected by many factors. Among them, social attributes cause people’s behaviors to be affected by the surrounding environment (3). Jinhua Liu (4) points out that the economic behaviors of residents in a group will be affected by others’ behaviors due to social interaction. China, as a relational society, has formed a social network with kinship, geography, and trust as the core for thousands of years. It is an important hub in the process of information acquisition and resource allocation, and exerts an important influence on individual economic decision-making. In the social network, the relationships that mainly affects individual behavior are called “strong social relationships,” and are composed of kinship and friendship. So, how do strong social ties influence the choice of long-term care patterns among middle-aged and older adults? What is the influence of different types of strong social relations on the long-term care patterns of middle-aged and older adults? In view of the above questions, this study uses the 2018 data of the China Health and Retirement Longitudinal Study (CHARLS) to explore the influence of strong social ties on the choice of care models of middle-aged and older adults in China. On a theoretical level, the paper investigates how certain social characteristics of an individual may affect their choice of long-term care setting and makes an attempt to explain how this can be the case. On the practical level, based on the theory and empirical results, this study proposes corresponding policy suggestions for the development of formal care patterns in order to further popularize social care and enhance China’s senior disability care system.

The structure of the rest of this study is as follows: the second section reviews the existing literature and puts forward the theoretical basis and research hypothesis. The third section describes the data sources, research samples, and statistical models. The fourth section includes descriptive statistics, regression analysis, robustness tests, and heterogeneity analysis of variables. Finally, the last section is the summary and recommendations.

## 2. Theoretical basis and research hypothesis

### 2.1. Theory of strong social relations

A social network, or social relationship network, was first proposed by Radcliffe Brown, who believed that a social network is a kind of social relationship that allows its members to maintain intimate contacts and is a concept of social capital. Academic circles mainly interpret social capital as “actual or potential resource collection, which consists of mutual default or recognition relations” (5). Based on the concept of social capital, the definition of social capital and social networks have been derived correspondingly, but, fundamentally, the discussion is still aimed at the relationship between individuals and their groups. Mark (6) argued that individuals are not completely independent when making decisions, and the social network they are in has a certain influence on their behavioral decisions. A large number of studies have proved that the deeper an individual is embedded in the social network, the greater the possibility of social participation. Social networks can not only help families disperse certain risk impacts and smooth household consumption, but also realize a certain degree of resource sharing and reduce transaction costs. At the same time, social networks can provide certain emotional support for their members and reduce the influence of negative psychological factors through mutual communication. In the social relations theory proposed by Mark (7), the social relations of residents are mainly divided into two categories: “strong social relations” and “weak social relations.” Among them, “strong social relationships” refer to the social networks with strong homogeneity and strong convergence of information exchanged between people. In China, Confucian traditional culture has exerted a far-reaching influence on social development for a long time, forming a social structure with friends and relatives as the core and an “acquaintance society” as the main feature (8). In ordinary life, families invest in strong social relationships through social interactions such as human relations or traditional customs and gatherings. For a long time without formal institutions, strong social relationships were the main channels for family members to seek help from negative impacts (9).

To sum up, strong social relations are the main social relations that affects the economic behavior of Chinese residents. Based on this, this article draws on the classification of existing literature (10) and divides strong social relations in China into the two major categories of kinship and friendship by using consanguinity, business relationships, and interest relationships as the main standards. Kinship is mainly based on consanguinity, including spouses, blood relatives, and in-laws (Article 1,045 of the Civil Code). On the other hand, friendship is based on business relationships and interest relationships, Friendship is a kind of non-blood relationship, its formation is mainly based on business and interest. In China, close friends will transfer funds such as gift money and rescue money in the process of communication (11).

#### 2.1.1. Kinship and choice of care model for older adults

In China, Kinship based on blood has always been an important way for families to spread risks. Fei (12) proposed that traditional Chinese society is a “differential pattern” based on clan groups and the network relationships centered on kinship. Everyone is in the center of the influence range exerted by his or her social network, and, at the same time, he/she is affiliated to the circle centered by people superior to himself/herself. Kinship implies the obligation of reciprocity, and mutual assistance is mainly carried out through gifts, loans, labor assistance, and other means, which plays a role in smoothing household expenditure and resisting family risks (13). By virtue of its reciprocity and altruism, kinship provides necessary help for the special moments in the life cycle of individuals in its social network, and has the function of risk transfer, such as the care or support services provided by kinship (14). At the same time, reciprocal and mutual assistance based on kinship can effectively deal with the problem of “moral hazards.” Good kinship is one of the important indicators of character evaluation in China (15), Instead of hoping for additional benefits, residents frequently turn to kinship to minimize their losses. Therefore, under the influence of family orientation, home care, which emphasizes “raising children for old age” and “mutual assistance by relatives,” has been the most important model of care for older adults in China for thousands of years. Even though the market economy and modern thinking have had a series of impacts on the traditional culture of the family (16), Chinese traditional parent–child feedback concepts of care for older adults still carries considerable weight among modern residents. Home care is still the preferred way of care for residents, and the mutual assistance and reciprocity generated by traditional kinship may have a certain substitution effect on formal care models such as institutional care.

But at the same time, kinship may have a certain positive impact on residents’ choice of formal care model. Weber once proposed that the foundation of all trust in China is obviously based on kinship or purely personal kinship. Kinship has the advantage of natural high trust and has a considerable aggregation effect. Through kinship, residents can transfer and share information resources, transmit and perceive risks, which may influence purchasing decisions for formal care. Based on the above analysis, this study proposes the following hypothesis:

*H1.1*: Kinship has a substitution effect on the choice of institutional care for middle-aged and older adults.

*H1.2*: Kinship can promote the choice of institutional care for middle-aged and older adults.

### 2.2. Friendship and older adult care model choice

As an important part of a strong social relationship, friendship has long been embedded in traditional Chinese culture and has a profound impact on residents’ economic life (17). In most existing studies, friends are defined as social relationships based on business ties and interest ties (18), which often include both ordinary friends based on interest ties, and colleagues, classmates, and friends based on business ties (19).

By virtue of its trust advantage, the information conveyed by strong social relationships is more likely to be adopted by residents (20), reducing the cost of information search and enhancing the participation in collective behaviors. Residents have social interactions with the network of friends through human relations such as “exchange of gifts,” which promote the degree of trust between them, so as to carry out high-quality information transmission and sharing. Liu and Du (21) used simulation experiments to find that disaster experiences in friends’ social networks would also encourage non-disaster groups to make plans for a rainy day. According to the existing literature, the paths that friend relationship affects individual behavior can be summarized into risk perception (22), imitation, and learning (23). Among these, the risk perception pathway refers to an individual’s understanding of risk information and obtaining risk perception through conversations with friends (24). Learning means that residents acquire socialized formal care information through friendship communication, learn relevant knowledge, and make choices independently (25). Imitation mainly refers to the behavior of residents simply imitating and copying a selection of peers without understanding relevant knowledge (26). Compared with other countries, formal care in China started later, and residents have relatively little understanding of the relevant information. Socialized formal care behavior transmits information in friendship through the above three paths, which may improve the participation rate of residents’ formal care behavior to a certain extent. Based on this, this study proposes the following hypothesis:

*H2.1*: Friendship can promote older adult’s choice of social care.

*H2.2*: Friendship has an inhibitory effect on older adult’s choice of social care.

## 3. Research methods

### 3.1. Sample selection

The empirical analysis of this study is based on the 2018 data of the China Health and Retirement Longitudinal Study (CHARLS). The China Health and Retirement Longitudinal Study aims to collect a set of high-quality microdata representing the households and individuals of middle-aged and older adults aged 45 and above in China to analyze the problem of population aging in China and promote interdisciplinary research on the problem of aging. The CHARLS national baseline survey was carried out in 2011, covering 150 county-level units, 450 village-level units, and 17,000 people in about 10,000 households. By the time the nationwide follow-up was completed in 2018, the sample had covered 19,000 respondents from 12,400 households, covering 11 aspects including personal information, health status, and medical insurance services. The access response rate and data quality of CHARLS are among the top of similar projects in the world, and the data has been widely used and recognized in the academic world.

By screening the data of CHARLS in 2018, this study limited the age of the sample Chinese middle-aged and older adult groups to 45–60 years old. At the same time, individual samples with key information missing and unavailable information were eliminated. Finally, 19,728 samples with relatively complete information were retained for empirical discussion, among which 5,698 urban samples and 14,030 rural samples were retained.

### 3.2. Variable description, data sources, and descriptive statistics

#### 3.2.1. Explained variables

This study treats the use status of middle-aged and older adult’s home and community care service in China as the explained variable and is expressed by care_all. According to question EH005_W4 in the 2018 CHARLS questionnaire: “Have you enjoyed the following home and community care services?,” the options include “day care center, nursing home, dining table for the older adults,” “regular medical check-up,” “home visit,” “family bed,” “community care,” “health management,” “recreational activities,” “other” and “none of the above,” In this paper, the first eight options are defined as choosing home and community aged care services and coded as “1 (formal care),” and the option “None of the above” is defined as choosing informal care and coded as “0 (informal care).”

#### 3.2.2. Explanatory variables

The core explanatory variable of this study is the strong social relations of Chinese residents. According to the two main hypotheses put forward in the theoretical basis, this study divides the core explanatory variables of strong social relations into kinship and friendship.

##### 3.2.2.1. Kinship

The core explanatory variable of H1 hypothesis is the relative relations of Chinese residents. According to the existing research, kinship is a relatively broad concept. This study draws on the existing literature and studies 5 groups of indicators in CHARLS 2018 (27), which are: “How much financial support did you or your spouse give/receive from your parents in the past year?,” “How much financial support have you or your spouse given/received from your children in the past year?,” “How much financial support did you or your spouse give/receive from your siblings in the past year?,” “How much financial support have you or your spouse given/received in the past year to other relatives or friends you do not live with who are not parents, children, or siblings?,” and “How much money have you or your spouse given/received in the past year to other relatives or friends you do not live with who are not parents, children, or siblings?” In this paper, the variable hosnet_R is constructed by combining the above 5 groups of indicator data and processing them logarithmically. Human exchange is a Chinese custom and tradition, which is usually embodied in mutual economic support in kinship relationships. The higher the amount and frequency of economic support, the closer the kinship relationship. Therefore, the construction variable hosnet_R can reflect the real situation of kinship relationships in strong social relationships to a certain extent.

##### 3.2.2.2. Friendship

In sociology, according to the “Granovetter theory of social relations,” close friends are often regarded as strong social relations together with family members and relatives (28). The definition of “friends” in the relevant literature is mostly based on business ties and interest ties (29). However, the scope of fun ties is relatively larger than business ties, and close work relationships are often included in the relationship of friends, which is difficult to distinguish (19). Therefore, this study defines the independent variable (friendship of Chinese residents) assumed by H2 as non-blood and non-in-law relationships based on interests or business relationships.

**Figure 3:**
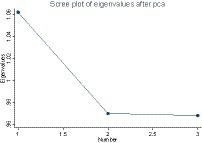
Rubble diagram of PCA.

As the definition of friendship is relatively virtual and broad, it is difficult to measure a single index in the questionnaire. Therefore, this study refers to the practice of Wang Xiaoquan (13) and selects three indicators of CHARLS 2018 data on the basis of considering both economic factors (social spending) and non-economic factors (social frequency), which are: “How much money did you or your spouse spend on catering for weddings and funerals, moving to a new house, newborns, children going to school and so on in the past year?,” “How many meals did your guests have at your home in the last week, calculated by person?” and “Have you engaged in the following social activities in the past month?.” Using the principal component analysis method (30), standardized data transformation through the idea of dimensionality reduction transformed multiple indicators into a relatively comprehensive friendship measurement index (30), which focuses on reflecting the degree of closeness between an individual and the friendship network, and is defined as hosnet_F. Firstly, KMO analysis was performed on the three selected groups of variables and the results showed that principal component analysis could be performed (see Table 1). According to the results of the principal component analysis in Table 2, the horizontal line at the point where the eigenvalue is equal to 1 of the gravel diagram (see Figure 1) is the cut-off point of the retained principal component, and the first principal component is selected to measure the friendship (Figure 3; Table 3).

**Table 1:** KMO test results of principal component analysis.

Item	KMO	Principal component
In the past year, how much did you or your spouse spend on catering for weddings and funerals, moving to a new house, a new baby, a child going to school, etc.?	0.5157	0.5715
How many meals did your guests have at your home in the last week, per person?	0.5150	0.5804
Have you had any of the following social activities in the past month?	0.5150	0.5801
Full sample	0.5152	-

**Table 2:** Results of principal component analysis of the friendship index.

Principal component	Eigenvalue	Proportion	Cumulative contribution rate
Comp1	1.06202	0.3540	0.3540
Comp2	0.96993	0.3233	0.6773
Comp3	0.96805	0.3227	1.0000

**Table 3:** Descriptive statistics of friendship indicators (principal component analysis).

Variables	Sample size	Mean	Max	Min	Standard deviation
hosnet_F	11,502	8.37e-09	36.8922	−0.6346	1.0305

#### 3.2.3. Control variables

In this study, a total of 10 control variables were set from the three levels of individual, family, and region, which are, respectively: (1) Personal characteristic variables, including age, gender, education level, marital status, health status, and social security; (2) family characteristic variables, which mainly refer to monthly household expenditure, household size, etc.; (3) regional characteristic variables, including urban dummy variables and provincial dummy variables. The geographical location and economic gap of different regions will affect residents’ choice of long-term care model.

The specific description of each variable in the model and some descriptive statistics are shown in Table 4.

**Table 4:** Descriptive statistics of main variables.

Metrics	Variables		Meaning	Mean	Description of value
Dependent variable		care_all	Use of home and community older adult care services	0.1975	1 = Chose formal care, 0 = Chose informal care
Independent variables		hosnet_R	Kinship	1.4961	Maximum = 14.0037, Minimum = 0, Standard deviation =1.4961
		hosnet_F	Friendship	8.37e-09	Maximum = 36.8922, Minimum = −0.6346, Standard deviation =1.0305
Control variables	Personal characteristics	Age	Age	64.8572	Maximum =121, Minimum =45, Standard deviation =10.2065
		Gender	Gender	0.4730	0 = Female, 1 = Male
		Edu	Education background	0.1269	0 = High school diploma or below, 1 = High school diploma or above
		Married	Marital status	0.8508	0 = Others, 1 = Married
		Health	State of health	0.2501	0 = Fair or bad, 1 = Good
		Insurance	Social Security	0.9697	0 = Uninsured, 1 = insured
	Family characteristics	L_expen	Household expenditure logarithm	7.1346	Maximum =11.9184, Minimum = −2.3026, Standard deviation =1.0272
		Family	Household size	7.1346	Maximum =20, Minimum =0, Standard deviation =1.8437
	Regional characteristics	Urban	Rural and urban dummy variable	0.2888	0 = Rural, 1 = Urban
		Province	Provincial dummy variable	0.3090	0 = Mid-west China, 1 = East China

### 3.3. Model setting and strategy

Logistic regression is the most widely used basic method in the field of statistical analysis, which is often used to simulate the probability of things happening. As the explained variable in this paper is care_all , which is a virtual binary variable, and compared with the Probit model, the regression coefficient of the logistic model is easier to explain its economic significance, so this study adopts the binary logistic model for analysis:


(1) $$\log\;it\left(P\right)=\ln\frac{P}{1-P}=\alpha +\sum_{i=1}^{n}\beta_{i}X_{i}$$

Based on the logistic regression model described above, two models were constructed with the same characteristics, including individual characteristics, family characteristics, and regional characteristics. The difference was that the explanatory variable of Model 1 was modeled using hosnet_R in strong social relations. Model 2 was modeled using the friendship hosnet_F in strong social relationships. The specific model is as follows:

Model 1:


(2) $$P\left(care\_all=1|hosnet\_R,X\right)=\varphi \left(\alpha +\beta_{1}hosnet\_R+\beta_{2}X+\varepsilon \right)$$

Model 2:


(3) $$P\left(care\_all=1|hosnet\_F,X\right)=\varphi \left(\alpha +\beta_{1}hosnet\_F+\beta_{2}X+\varepsilon \right)$$

where care_all=1 represents the probability of respondents choosing the formal care model, and care_all=0 represents the probability of respondents choosing informal care; hosnet_R and hosnet_F are the core explanatory variables of this study, kinship and friendship; X is the relevant control variables that influence the respondents’ choice of long-term care model, including personal characteristics, family characteristics, and regional characteristics; and ε represents the random error term.

## 4. Research results and analysis

### 4.1. Sample characteristics and correlation analysis

By showing the answers to the questions in the CHARLS 2018 questionnaire, this study presents some descriptive statistics of the respondents’ personal characteristics, family characteristics, and regional characteristics. As shown in Table 5, the proportion of surveyed groups choosing a formal care model accounted for only 19.75%, indicating that the formal care model in China still has space to develop. Comparing the group that chose the formal care model with the group that did not, the average age of the group that chose the formal care model was 1.69 years older than the group that did not choose the formal care model, indicating that the older the group, the greater the demand for formal care, and also indicating that there may be a certain adverse selection tendency for the formal care model. At the same time, comparing the social security level of the group that chose the formal care model and the group that did not shows that the group with social security was more inclined to choose the formal care model.

**Table 5:** Individual characteristics and descriptive statistics of samples.

Chose formal care model	Sample proportion	Average age	Household size	Proportion with high school education or above
Yes	19.75%	73.57	3.05	8.82%
No	80.25%	71.88	3.08	9.58%
**Chose formal care model**	**Proportion of urban areas**	**Proportion of east China**	**Proportion of social security**	**Proportion of healthy population**
Yes	27.06%	37.07%	98.02%	21.95%
No	26.69%	30.07%	96.71%	20.79%

From the perspective of the regional characteristics of the respondents, compared with the residents in rural areas, the proportion of urban residents choosing the formal care model was higher than that of the former. The proportion of residents in east China choosing the formal care model was higher than that in mid-west China, which indicates to some extent that the choice of long-term care model may be affected by the local formal care physical facilities and economic development level. At the same time, regional differences may lead to differences in the concept of old-age care and also have an impact on the choice of formal care model by middle-aged and older adults.

### 4.2. Analysis of regression results

The baseline regression results of kinship and friendship are shown in Table 6. In the strong social relationship, column (1) only carries out regression for the core explanatory variable hosnet_R , while all variables are controlled in column (2). The estimated odds ratio between the two is small and in the same direction, which can eliminate the problem of missing variables to a certain extent. According to the results of column (1) and column (2) of kinship relations, hosnet_R has a positive promoting effect on the selection of the formal care model for middle-aged and older adults, which verifies hypothesis H1.2 and excludes hypothesis H1.1. This shows that the increasingly compact and dispersed family structure in China can no longer meet the increasing long-term care demands of middle-aged and older adults. Residents have to choose other ways to disperse the pressure of family care, which sparks the demand for the formal care model, demonstrating that family relationships have a “squeezing effect” on formal care for middle-aged and older adults. The above results indicate that residents are gradually realizing that the combination of the traditional “child-rearing” family care model and the socialized formal care model can better meet the long-term care needs of middle-aged and older adults.

**Table 6:** Results of baseline regression.

Variables		Kinship		Friendship	
		(1)	(2)	(1)	(2)
Personal characteristics	hosnet_R	1.063^***^ (2.80)	1.091^***^ (3.55)	-	-
	hosnet_F	-	-	1.147^***^ (4.16)	1.128^***^ (3.43)
	Age		1.03^6***^ (7.28)		1.036^***^ (7.52)
	Gender		1.048		1.045
	Edu		0.914		0.900
	Married		0.881^*^ (−1.61)		0.934
	Health		1.069		1.065
	Insurance		1.736^**^ (2.47)		1.684^**^ (2.51)
Family characteristics	L_expen		1.032		1.038
	Family		1.010		1.000
Regional characteristics	Urban		1.007		1.070
	Province		1.318^***^ (3.94)		1.403^***^ (5.01)
Sample size		6,273	5,658	6,778	6,054
Adjusted R-squared		0.0012	0.0168	0.0024	0.0177

Z-values in parentheses. ^***^, ^**^, and ^*^ are significant at the level of 1%, 5%, and 10%, respectively.

The results of column (1) and column (2) of friendship indicate that hosnet_F has a positive impact on the choice of the formal care model among Chinese middle-aged and older adults, which supports hypothesis H2.1 but excludes hypothesis H2.2. The baseline regression results show that the transmission and perception of risk by residents communicating with friends will increase the possibility of middle-aged and older adults choosing the formal care model. At the same time, residents may transmit the behavior of choosing the formal care model among groups by imitating the behavior of friends, which is conducive to the improvement of the selection rate of the formal care model.

Meanwhile, the baseline regression empirical results show that “age” has a significant positive impact on the choice of the formal care model for middle-aged and older adults, indicating that, with the increase of age, the risk of disability and dementia of residents gradually increases, which leads to a gradual increase in the demand for the formal care model. According to the empirical results, “social security” also has a significant positive impact on the explained variable ( care_all ), and the groups with social security are more inclined to choose the formal care model. In terms of regional characteristics, samples from east China have a significant positive impact on care_all . The economic development level of east China is higher than that of mid-west China, and the physical facilities and institutions related to formal care are better and more popular, which is conducive to middle-aged and older adults choosing the formal care model.

### 4.3. Robustness test

As for the robustness of the above results, this study adopted two methods to test them. The robustness test results are shown in Table 7: first, regression is conducted by replacing the core explanatory variables and the descriptive statistics of the replacement variables are shown in Table 8. In terms of kinship, this study drew on the practice of Wang Peihui (30) and selected 10 sets of data from CHARLS 2018, including “How much financial support did you or your spouse give/receive to other relatives and friends who are not parents, children or siblings that you do not live with in the past year?” and “How much money have you or your spouse given/received in the past year to other relatives or friends you do not live with who are not parents, children, or siblings?” and standardized the variables after the sum to build the kinship indicator clan in the robustness test. The method of normalization is to assign the value of 1 to the sum of economic transactions and the rest to 0. For the relationship between friends, this study selected the question “How often have you made the above social contact in the past month?” from CHARLS 2018 data. The friend relation index in the robustness test is defined as the frequency of communication between an individual and his friends. By repeating the previous regression steps with the substitution variables, while keeping the other conditions unchanged, the results obtained were consistent with the original results.

**Table 7:** Robustness test of strong social relationship influence.

Variables	Logit (1)	Logit (2)	Probit (1)	Probit (2)
Clan	1.114^**^ (1.93)	-	-	-
Friend	-	1.111^***^ (4.38)	-	-
hosnet_R	-	-	0.0503^***^ (3.56)	-
hosnet_F	-	-	-	0.0720^***^ (3.42)
Other variables (same as Table 6)	Yes	Yes	Yes	Yes
Sample size	9,630	9,630	5,638	6,054
Adjusted R-squared	0.0172	0.0188	0.0171	0.0188

Z-values in parentheses. ^***^, ^**^, and ^*^ are significant at the level of 1%, 5%, and 10%, respectively.

**Table 8:** Statistical characteristics of the replacement explanatory variables in the robustness test of strong social relations.

Variables			Sample size	Mean	Standard deviation	Min	Max
Independent variables	Kinship	Clan	19,828	0.5460	0.4979	0	1
	Friendship	Friend	19,828	0.7048	1.1199	0	6

Secondly, this paper uses the probit model to re-estimate the impact of strong social ties on the long-term care patterns of middle-aged and older adults. Under the condition that the control core explanatory variables and other variables remain unchanged, the results obtained by changing the regression model were basically consistent with the benchmark regression, and the core explanatory variable coefficients of kinship and friendship in the probit model were all significant at the 1% level.

### 4.4. Heterogeneity analysis: regional and group differences

Due to the vast size of China, the economic development levels and cultural customs of different areas where residents are relatively different leads to certain differences in the strong social relations, which affects middle-aged and older adults’ choice of long-term care model. Therefore, this study analyzed the heterogeneity of provinces and urban and rural areas, and the regression results are shown in Table 9.

**Table 9:** Results of heterogeneity analysis.

Variables	Provinces		Urban and rural	
	East China	Mid-west China	Urban	Rural
hosnet_R	1.043 (0.99)	1.121^***^ (3.75)	1.064 (1.30)	1.102^***^ (3.37)
hosnet_F	1.094 (1.31)	1.143^***^ (3.24)	1.126^*^ (1.75)	1.131^***^ (2.97)
Other variables	Yes	Yes	Yes	Yes
Sample size	1744	3,914	1,506	4,152
Adjusted R-squared	0.0102	0.0204	0.0147	0.0201

Z-values in parentheses. ^***^, ^**^, and ^*^ are significant at the level of 1%, 5%, and 10%, respectively.

From the perspective of regional characteristics, the positive effect of hosnet_R on the selection of the formal care model care_all was more significant in the mid-west and rural areas, but had no significant effect on east China and urban areas. The concept of clan and kinship was stronger in rural areas and mid-west China. Kinship had a greater impact on local residents’ choice of daily behavior and thus on their choice of long-term care model.

For hosnet_F , the choice of care_all was significantly affected by friendship in mid-west China, while not significantly affected by the relationship of friends in east China. This indicates that the social network dominated by strong social ties still plays an important role in the life of residents in mid-west China. From the perspective of urban and rural areas, friendship had a positive and significant impact on both urban and rural residents.

### 4.5. Analysis of influence mechanism: “SIRS Infectious Disease Model”

**Figure 4:**
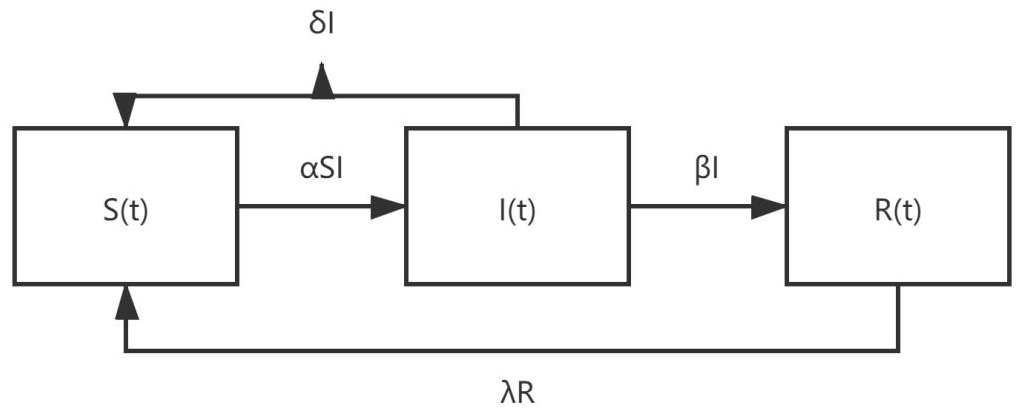
Dynamic model of the formal care market and strong social relationships.

Referring to the existing literature, this paper adopted the “SIRS infectious disease model” to explore the influence of strong social relations on the choice of long-term care model for middle-aged and older adults (31). The SIRS infectious disease model mainly describes the abstract process of information transmission. S, I, and R stand for susceptible, infective, and removed, respectively. In this paper, under the effect of strong social relations, assuming that the economy is a closed economy and the total number of people at time t is N(t), the overall selection of the market for long-term care models can be regarded as people who did not choose formal care model S(t), people who perceived risk but did not choose formal care model I(t), and people who chose formal care model R(t). α represents the strength of strong social ties and β represents the learning and imitation behaviors caused by strong social relationships. The group S(t) who did not choose the formal care model and the risk-sensing group I(t) transmitted information through social interactions to stimulate their own risk perception. Part of the risk-sensing group βI chose formal care behaviors through imitation and learning behaviors. δI indicates that although the risk-sensing population I(t) senses the risk, it still does not choose the formal care model. Under the action of time t, it returns to S(t). λR refers to the group of people who chose the formal care model before and then did not choose to return to S(t), and the dynamic change of the formal care market is shown in Figure 2. At the same time, it is assumed that: (1) the population remains a constant, i.e., N(t)≡N (2) each population contact must have a certain infectivity, that is, α, β, λ, and δ are greater than 0; (3) in the initial state, S>>I>R . According to the dynamic transformation of the formal care market, the dynamic equation is constructed (Figure 4).


(4) $$\frac{dS\left(t\right)}{dt}=-\alpha S\left(t\right)I\left(t\right)+\lambda R\left(t\right)+\delta I\left(t\right)$$

(5) $$\frac{dI\left(t\right)}{dt}=\alpha S\left(t\right)I\left(t\right)-\beta I\left(t\right)-\delta I\left(t\right)$$

(6) $$\frac{dR\left(t\right)}{dt}=\beta I\left(t\right)-\lambda R\left(t\right)$$

Since the total population N of this economy is assumed to be constant in this study, the equation R = N-S-I can be obtained as follows:


(7) $$\frac{dS\left(t\right)}{dt}=-\alpha S\left(t\right)I\left(t\right)+\lambda \left[N-S\left(t\right)-I\left(t\right)\right]+\delta I\left(t\right)$$

(8) $$\frac{dI\left(t\right)}{dt}=\left[\alpha S\left(t\right)-\beta -\delta \right]I\left(t\right)$$

Set the above two equations equal to 0 and the above steady-state value can be obtained as:


(9) $$S^{*}=\frac{\beta +\delta }{\alpha }$$

(10) $$I^{*}=\frac{\alpha \lambda N-\left(\beta +\delta \right)\lambda }{\alpha \left(\beta +\lambda \right)}=\frac{\lambda N}{\beta +\lambda }-\frac{\left(\beta +\delta \right)\lambda }{\alpha \left(\beta +\lambda \right)}$$

(11) $$R^{*}=\frac{\beta \left(N\alpha -\beta -\delta \right)}{\alpha \left(\lambda +\beta \right)}$$

It can be seen from the steady-state value that, at $\;S^{*}$ , each group reaches an equilibrium state in the formal care market. Among them, the group $S^{*}$ that does not choose formal care will decrease with the increase of interaction frequency α between groups, which means that more people switch to risk perception group $I^{*}$ and choose formal care group $R^{*}$ indicating that strong social relationships will affect the group’s demand for the formal care model.

In this paper, the “SIRS infectious disease model” is constructed to clarify the influence mechanism between strong social ties and the formal care model. In the empirical regression, questions such as “Do you participate in supplementary medical insurance?” in the 2018 CHARLS questionnaire were selected to construct the medical transfer payment index medical. The analysis results are shown in Table 10. The regression results of model (1) and model (2) show that the group that attaches more importance to strong social ties will increase medical transfer payments, thus increasing the demand for the formal care model. In model (3), sample individuals were limited to healthy individuals (heath = 1), and the results were still significant, indicating that even though individual residents themselves did not suffer from disability risk, risk information transmitted by strong social relationships would stimulate their own needs for the formal care model.

**Table 10:** Analysis results of influence mechanism.

Variables	Medical (1)	care_all (2)	care_all (3)
Medical	–	1.823^***^ (4.58)	1.402^*^ (1.76)
hosnet_R	1.004 (0.13)	1.097^***^ (3.65)	1.099^**^ (1.76)
Other variables	Yes	Yes	Yes
Sample size	9,181	5,403	1,124
Adjusted R-squared	0.0403	0.0208	0.0281
Variables	medical (1)	care_all (2)	care_all (3)
Medical	–	1.787^***^ (4.56)	1.444^*^ (1.95)
hosnet_F	1.082^**^ (2.50)	1.123^***^ (3.20)	1.220^**^ (2.33)
Other variables	Yes	Yes	Yes
Sample size	9,832	5,766	1,200
Adjusted R-squared	0.0408	0.0209	0.0351

Z-values in parentheses. ^***^, ^**^, and ^*^ are significant at the level of 1%, 5%, and 10%, respectively.

## 5. Conclusion and discussion

Faced with the increasingly severe problem of population aging in China and the phenomenon of smaller family sizes and fewer children, the development of the formal care model plays an important role in alleviating the pressure of care for middle-aged and older adults. The research results show that strong social relations, as an important “reference point” in residents’ daily life, play an important role in residents’ behaviors and choices, and the relationships with relatives and friends will affect the behavioral decisions of middle-aged and older adults in China, which is consistent with previous research results (16, 32), and will have a certain impact on their choice of care model. Strong social relationships such as kinship and friendship tend to contribute to the choice of formal care models among Chinese middle-aged and older adults through transmission mechanisms such as risk perceptions (3). At the same time, Due to the large regional differences and the special urban-rural dual structure in China, there are also obvious regional and urban-rural differences in the results: mid-west China and rural areas are more significantly influenced by strong social ties, while east China and urban areas do not show significant results.

Based on the above results, this study puts forward the following recommendations for the development of the formal care model: firstly, it should be clear that there is a certain competition and cooperation relationship between strong social relations and the formal care model in terms of risk dispersion and transfer. Influenced by the traditional concepts of “raising children for old age” and “mutual assistance from relatives,” the family care and old-age care model is still the first choice for Chinese residents. China should explore more innovative formal care models such as home care and community joint care, and focus on the development of neighborhood (township) and urban and rural community pension service networks, so as to realize the gradual transition from family care to formal care. Secondly, the establishment of good and strong social network relations should be promoted, using strong social relations to promote formal care model. The study and imitation effect of relative relations and friends produce important influence on the choice of daily behavior of Chinese residents, so China should encourage good social relations, and encourage relatives and friends to help each other. Through propagating and popularizing formal care knowledge among strong social networks, the information and education of older adult health knowledge in urban and rural communities is strengthened, the health literacy of older adults is improved, and the formal care model is promoted. Furthermore, through the establishment of a harmonious and friendly strong social network, the old-age care system to provide informal protection for relieving the care pressure of middle-aged and older adults is enriched.

The innovation of this study lies in the fact that, starting from the “differential pattern” of traditional Chinese relationships, kinship based on blood relationships and friendship based on business relationships and interest relationships were selected as “strong social relationships.” This paper explains the influence of different strong social relations on residents’ choice of care mode, and tries to give an explanation. It is a supplement to the previous research and provides a reference for the existing research on how other social relations affect the choice of residents’ care model. At the same time, this study still has some shortcomings. Due to data limitations, most databases are only updated to 2018, and some variables are not continuous, so they cannot be sorted into microscopic panel data, which is also the limitation faced by most empirical papers at present. Furthermore, there are many kinds of interpersonal relationships, so it is difficult to cover them all. This study can only focus on the relative major strong social relationships, and the demonstration of secondary relationships needs to be further explored.

## Data availability statement

The original contributions presented in the study are included in the article/Supplementary material, further inquiries can be directed to the corresponding author.

## Author contributions

Zhao X. contributed to the conception and design of the study, organized the database, performed the statistical analysis, and wrote the first draft of the manuscript. Xing H. and Jing T. validated the statistical analysis and reviewed, edited, and revised sections of the manuscript. Bao C. and Zhan L. contributed to manuscript methodology and software, revision, and read and approved the submitted version of the manuscript.

## Funding

This work was supported by the Ministry of Education of Humanities and Social Science Project, “Research on the ‘In-home Supportive’ Endowment Pattern for Disabled Elders in Chinese Rural Area under the Background of Population Aging and Moving,” 2016, project number: 16YJA840004, Project Leader: TJ.

## Conflict of interest

The authors declare that the research was conducted in the absence of any commercial or financial relationships that could be construed as a potential conflict of interest.

## Publisher’s note

All claims expressed in this article are solely those of the authors and do not necessarily represent those of their affiliated organizations, or those of the publisher, the editors and the reviewers. Any product that may be evaluated in this article, or claim that may be made by its manufacturer, is not guaranteed or endorsed by the publisher.

## Footnotes

^1^National Bureau of Statistics of China. *The Seventh National Population Census.* [EB/OL]. http://www.stats.gov.cn/ztjc/zdtjgz/zgrkpc/dqcrkpc/.

^2^China Aging Working Committee Office. The survey of disabled elderly in *the Survey Report on the Living Conditions of Urban and Rural Elderly People in China (2018).*

^3^CEIC. http://www.ceicdata.com/zh-hans/china/population-no-of-person-per-household.

^4^The "9,073" elderly care pattern refers to that about 90% of the elderly live at home, 7% rely on community support for elderly care, and 3% live in institutions for elderly care.

^5^Department of Aging Health of the National Health Commission of the People’s Republic of China. Policy interpretation of *the Guiding Opinions on Establishing and Improving the Health Service System for the Elderly.* [EB/OL]. http://www.nhc.gov.cn/lljks/s7786/201911/4cbecd7450694416a181f9b37e92.shtml,2019.11.01.

^6^The State Council of the People’s Republic of China. *Opinions on Strengthening the Work on Aging in the New Era.* [EB/OL]. http://www.mohrss.gov.cn/SYrlzyhshbzb/dongtaixinwen/shizhengyaowen/202111/t20211125_428653.html.

^7^"421" family pattern refers to a family consisting of four older adults people, a couple and a child.
